# CAR-T State of the Art and Future Challenges, A Regulatory Perspective

**DOI:** 10.3390/ijms241411803

**Published:** 2023-07-22

**Authors:** Lorenzo Giorgioni, Alessandra Ambrosone, Maria Francesca Cometa, Anna Laura Salvati, Armando Magrelli

**Affiliations:** 1 Faculty of Physiology and Pharmacology “V. Erspamer”, Sapienza Università di Roma, 00185 Rome, Italy; lorenzo.giorgioni@uniroma1.it; 2 Faculty of Medicine and Pharmacy, Sapienza Università di Roma, 00185 Rome, Italy; ambrosone.2025019@studenti.uniroma1.it; 3 National Center for Drug Research and Evaluation, Istituto Superiore di Sanità, 00161 Rome, Italy; mariafrancesca.cometa@iss.it; 4 Faculty of Pharmacy, Tor Vergata University of Rome, 00133 Rome, Italy; mgrrnd01@uniroma2.it

**Keywords:** CAR-T, EMA, rare diseases, ATMP, hospital exemption, compassionate use

## Abstract

This review is an outlook on CAR-T development up to the beginning of 2023, with a special focus on the European landscape and its regulatory field, highlighting the main features and limitations affecting this innovative therapy in cancer treatment. We analysed the current state of the art in the EU and set out a showcase of the field’s potential advancements in the coming years. For this analysis, the data used came from the available scientific literature as well as from the European Medicines Agency and from clinical trial databases. The latter were investigated to query the studies on CAR-Ts that are active and/or relevant to the review process. As of this writing, CAR-Ts have started to move past the “ceiling” of third-line treatment with positive results in comparison trials with the Standard of Care (SoC). One such example is the trial Zuma-7 (NCT03391466), which resulted in approval of CAR-T products (Yescarta™) for second-line treatment, a crucial achievement for the field which can increase the use of this type of therapy. Despite exciting results in clinical trials, limitations are still many: they regard access, production, duration of response, resistance, safety, overall efficacy, and cost mitigation strategies. Nonetheless, CAR-T constructs are becoming more diverse, and the technology is starting to produce some remarkable results in treating diseases other than cancer.

## 1. Introduction

Chimeric Antigen Receptor (CAR)-based therapies represent a significant development in immunotherapy, since they have the potential to be effective in relapsed and refractory (r/r) disease, where efficacy of other therapies is lower. They could also virtually be safer and more effective than traditional chemotherapy, though at the moment they present several unsolved limitations. CAR-T cell therapies are ATMPs and all those currently authorised are orphan drugs. This means they are complex medicinal products, made of biological materials engineered with cutting-edge technologies, currently indicated only for rare diseases, thus being subject to a composite regulatory framework.

Looking at the European pharmaceutical regulatory landscape, CAR-engineered medicinal products are specifically regulated through the following: The Advanced Therapies Regulation (EC) No. 1394/2007, which had the objective to discipline Advanced Therapy Medicinal Products (ATMPs) [1] in the EU, as well as the Orphan Medicines Regulation (EC) No. 141/2000 [2], complemented by the (EC) Regulation Number 847/2000 of the European Parliament and Council, which had the objective of rewarding interest in small patient populations neglected by the pharma industry due to unsustainable returns. These Regulations are presently under revision, so the landscape might change in the future [3].

Other pieces of legislation relevant to this review are those respectively establishing the Hospital Exemption (HE) and Compassionate Use (CU) in Europe. The former describes a regulatory option, foreseen by Article 28 of Regulation (EC) No 1394/2007 amending Article 3 of Directive 2001/83/EC, based on which any ATMP can be prepared as an individual medical prescription for an individual patient (named-based) on a non-routine basis, under the exclusive professional responsibility of a medical practitioner for the treatment of severe, disabling, or life-threatening conditions [1]. The latter (CU) was established by Article 83 of Regulation (EC) No 726/2004 and allows the use of investigational medicinal products in groups of patients affected by diseases with no satisfactory therapies who cannot enter clinical trials [4]. 

In order to provide an updated picture of CAR-Ts state of the art, together with developments most likely to be achieved in the near- and mid-term, as well as insights on what is currently possible and how the regulatory sector is enabling these products to reach the clinical setting, several sources and existing projects and initiatives have been considered for this review, namely: The EU Clinical Trials Register (clinicaltrialsregister.eu), supplemented by the US database of privately and publicly funded clinical studies (ClinicalTrials.gov); the EMA website’s EPAR Repository, to obtain the latest updates on information and indications of EU-authorised CAR-T medicinal products; the lists of medicinal products awarded with the PRIME scheme, as well as relevant examples of HE and CU to look at possible approaches for future Marketing Authorization Applications (MAAs). 

From a structural perspective, a CAR is an artificial protein expressed on the surface of an immune cell, encoded by a transgene introduced via a variety of methods, most commonly transfection via viral vectors. The majority of immune cells presently engineered belong to the cellular component of the adaptive immune system (CD4+ and/or CD8+ T cells), but engineering of other families of immune cells, especially those belonging to the innate immune system like Natural-Killer (NK) [5] and Macrophage (M), [6] have also been reported. 

All approved CAR-T products bear a chimeric antigen receptor composed of an antigen-binding domain typical of antibodies, a co-stimulatory factor from the T cell’s surface, and a signalling domain found in T-cell receptors (TCR), all deriving from different sources, hence the name ‘chimeric’. In a process of innovation already visible today and reported by other authors [7], this structure will likely become more complex and diverse in the future, with different proteins being used to fill the role of each domain, with the aim to increase safety and efficacy. 

Up to the first promising results from clinical trials in 2014, the participation in CAR-T research was scarce, but in recent years, Chimeric Antigen Receptor T cells have demonstrated to be an efficacious means to achieve enhanced survival in haematological malignancies and presented enough benefit to warrant marketing authorisations for several of the investigated medicinal products. These drugs are showing a very positive risk/benefit balance, with benefits continuing to improve as safety is enhanced by new construct designs and protocols to deal with treatment complications.

### CAR-T Cells Generation

**Figure 1:**
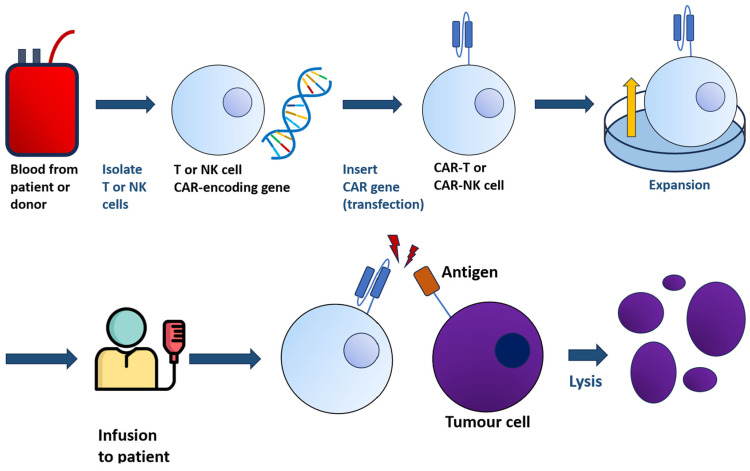
From a sample of blood, either from patients or donors, T or NK cells are harvested and transfected with the gene encoding CAR. The resulting CAR-T/-NK cells, bearing the chimeric receptor, are administered to patients. The interaction between CAR-T/-NK cells with tumour cells will lead to lysis.

The general principle of CAR-T-cell therapy is summarised in Figure 1.

**Figure 2:**
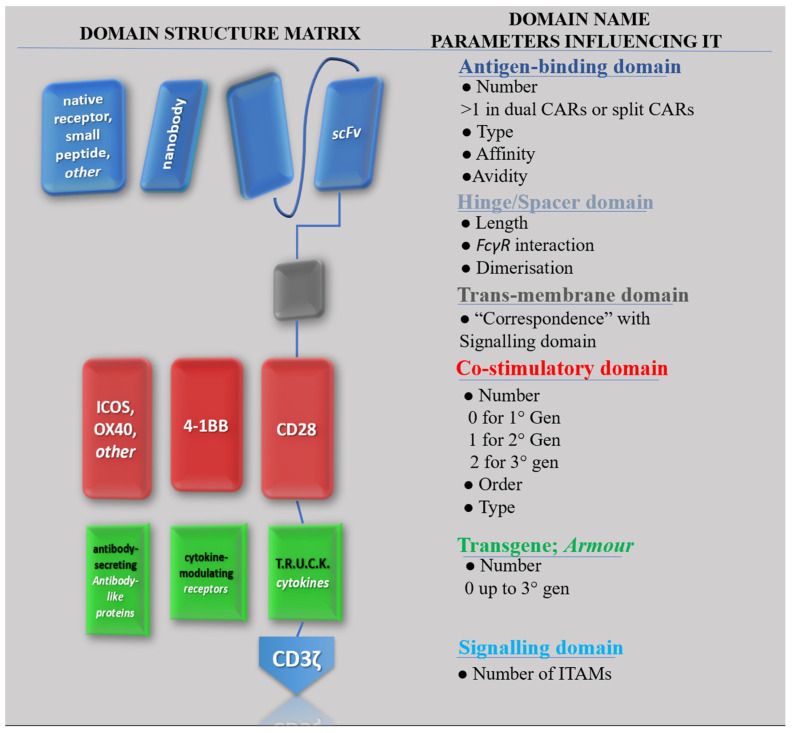
All possible CAR configurations with depictions of possible domains, their name, and the most relevant parameters influencing their activity. Proteins in rows on the left are potential candidates to fill the role of the list of domains on the right.

In CAR-T design, domains are sometimes referred to as modules [8]. This emphasises the technology’s modular character, with a virtually endless number of possible combinations of different domains making up the construct [9]. This concept of the “structural matrix” is exemplified in Figure 2, which aims to represent the most general possible structure for a CAR construct. Although in a “typical” CAR, the role of antigen-binding domain is filled by an antibody-derived scFv, other options reported in literature are Nanobodies [10], truncated versions of an scFv mainly featuring the heavy chains (VH), or native receptors, or even small peptides like the so-called peptide-centric [11] and SPEAR [12] CAR-Ts designs. Under each domain, parameters which influence its activity are indicated. For instance, the antigen-binding domain activity is affected by the number of “domains”, which can vary for specific constructs (e.g., dual CAR-T share two domains), but it is also influenced by the type of protein chosen to derive the antigen-binding domain, by its affinity and/or avidity for the antigen, or by the interaction of different CAR constructs’ affinities on a single CAR-T-cell surface. Each property originates different behaviours and cytologic phenomena which overall may influence the behaviour of the CAR in vivo, thus impacting its safety and efficacy profile [13].

## 2. CAR-T-Cell Therapies Approved in the EU

To describe the regulatory activity conducted by the EMA (European Medicines Agency) in terms of marketing authorisations, the online repository for EPARs (European Public Assessment Reports) (https://www.ema.europa.eu/en/medicines/whatwe-publish-when/european-public-assessment-reports-background-context, accessed 23 May 2023) was consulted. EPARs publicly report the positive opinion from EMA’s CHMP (Committee for the Human use of Medicinal Products) on a medicinal product, that subsequently led to its authorisation by the European Commission. Over the past few years, European marketing authorisations have significantly increased in terms of number, and the time to complete the process has been shortened, closely following US FDA (Food and Drug Administration) approvals. In addition, increase in indications for several medicinal products signals the growing use and interest in this therapeutic approach. Currently, six products have been authorised in the EU and thirteen different indications were evaluated by the CHMP (Table 1).

### 2.1. Regulatory Pathways to CAR-T Approvals

Several innovative regulatory pathways have been recently established and used in the context of the European pharmaceutical legislation for increasing access to these treatments. These pathways range from fast-track procedures, which tend to expedite their regulatory evaluation and increase the likelihood of reaching an approval, to newly adapted initiatives like the “PRIME scheme”, followed by “Hospital Extension” and “Compassionate Use”. 

#### 2.1.1. PRIME Scheme

PRIME is an acronym standing for “Priority Medicines”. It is a scheme first introduced in 2016 to specifically “enhance support for the development of medicines that target an unmet medical need” (https://www.ema.europa.eu/en/human-regulatory/research-development/prime-priority-medicines, accessed 23 May 2023). The scheme provides the ground for an accelerated assessment by the EMA, as well as regulatory support to the sponsor in overcoming hurdles encountered during clinical trials. 

PRIME is tied to CAR-T development from its beginnings: Kymriah and Yescarta, the first two CAR-T-cell therapies ever approved, were the first therapies granted with the PRIME status; but the connection with PRIME is not limited to these initial market approvals, as all the CAR-Ts currently authorised have been at some point awarded the PRIME scheme. As such, the PRIME scheme is considered to provide a significant view of those products that are likely to be approved in EU, in the near- and mid-term.

Out of the 16 medicinal products that were granted the PRIME designation up to 23 May 2023 and have completed the evaluation process, eight (8) were CAR-Ts (Table 2) and their therapeutic area was Oncology; six (6) of them have subsequently received marketing approval, while 2 were withdrawn. Data supporting the award of PRIME status to each of these requests were both Non-clinical as well as Early (Exploratory) Clinical.

Table 3 summarises all the CAR-Ts still under development, as extracted from the complete list of medicinal products currently awarded with the PRIME scheme. All these medicinal products are intended to treat oncological conditions, and four (4) of them target antigens present on solid cancers (Lete-Cel [14], ADP-A2M4 [15], BNT211 [16], CT041 [17]). Data supporting the PRIME status for these developments were both Non-clinical as well as Early (Exploratory) Clinical. 

It is important to note how, among the drugs in this list, some present constructs are not “typical”, in that they do not adhere to the description previously given and use an antibody-derived antigen-binding domain.

These “atypical” designs, specifically MB-CART2019.1 and ADP-A2M4, feature a dual-CAR [18] and a SPEAR CAR [19], respectively. The presence at this stage of non-conventional CAR constructs is an indication that the efforts underway to bridge the gap in efficacy in this field of therapy are an endeavour which requires increasingly innovative approaches.

Of note, given the successful experience with PRIME in improving and accelerating access to innovative medical products, this scheme is considered one of the key regulatory tools to be strengthened in the new proposal of the European pharmaceutical legislation [3].

#### 2.1.2. Hospital Exemption (HE)

Other regulatory paths where CAR-T candidates for future MAAs may be found are those that enable real-world use of the product in a clinical setting before approval. The major options in this regard in the EU are Hospital Exemption and Compassionate Use programs. 

In Italy, the hospital exemption is implemented through the authorisation procedure for medicines prepared on a non-repetitive basis, regulated through the Ministry of Health Decree of 16 January 2015. By querying the Italian Hospital Exemption database (ISS-AIFA) starting from 2021, twenty-six (26) CAR-T cell-based treatments were retrieved that have been authorised for patients with B precursor acute lymphoblastic leukaemia, neuroblastoma, and lupus erythematosus. Among the authorised treatments, twenty (20) involve paediatric patients (<18 years old) as shown in Table 4. 

The products were almost all allogeneic CAR-Ts obtained from HLA-identical donors, being in some cases members of the patients’ family. Fifteen (15) out of the twenty-six (26) cases used reference protocols similar to those used in phase I and I/II clinical trials, meaning they were already approved for experimental use.

Of note, despite the presence on the market of licensed products, HE has been successfully used to target patients otherwise excluded from any type of treatment.

In the case of Kymriah™, which was suitable for patients with relapsed disease and/or refractory to conventional treatments, some common limitations to its use (insufficient white blood cells quality or count to produce the autologous product) led to the demand for allogeneic CD19-CAR-T cells for relapsed and/or refractory B-cell acute lymphoblastic leukaemia (ALL) under HE. The most frequent cases submitted under HE included relapsed patients already undergoing heavily lymphodepleting chemotherapy cycles or treated with HSCT.

Similarly, with reference to the application of CAR-T cells in the neuroblastoma indication, an innovative product that uses an inducible iC9 caspase to control the possible CRS due to CAR-T therapy was used on twenty-seven (27) patients aged 1 to 25 years and the extremely promising results were published in April 2023 [20]. However, the closing of the recruitment phase excluded other possible treatment of eligible candidates from the study, and the HE application was undertaken to ensure patients with refractory and relapsed disease a viable therapeutic alternative. 

As a general limitation of this analysis, it has to be underlined that products administered throughout Hospital Exemption in EU are under the supervision of the corresponding national competent authority of each Member State (MS), after careful risk–benefit analysis of the medicinal product. The lack of a centralised register for HE in EU makes it difficult to retrieve information from each MS. However, reports may be found by looking at the available literature for studies on CAR-T HE use. As an example, the product ARI-0001 or CART19-BE-01, currently included in the list of PRIME medicines, is an anti-CD19 CAR against B-cell malignancies, which was approved by the Spanish Agency of Medicines and Medical Devices (AEMPS) under HE to treat adult patients (>25 years old) with r/r ALL [21] in 2021. The rationale for approval regarded the characteristics of this illness, which is rare, life-threatening, and presents few viable therapeutic alternatives in the r/r setting, thus respecting the criteria for HE requests. 

Safety and efficacy of ARI-0001 have been assessed in a study conducted together with the paediatric hospital Sant Joan de Déu (Barcelona), with similar results with respect to those of other products [21,22]. ARI-0001 is used in a Phase 2 study in adult patients with R/R CD19+ ALL (NCT04778579) due to complete in 2024. 

Also, in this case, HE constituted a valid alternative path foreseen by the European ATMP Regulation [1] to enhance patients’ access to advanced therapies, whereby evidence can be provided on the quality, safety, and efficacy profiles of products, even if manufactured under “non-routine” conditions.

#### 2.1.3. Compassionate Use (CU)

Compassionate Use [4] programs were searched for those drugs where public reports were available. The product chosen as an example for this regulatory option is not in the current list of PRIME medicines: it is called MB-CART19.1 [23], it targets CD19, and has been used against an autoimmune disease known as Systemic Lupus Erythematosus (SLE) [24,25] in Germany. It was offered to patients according to the Arzneimittelgesetz (German Drug Law), paragraph 21/2, and approved by the German Federal Institute for Drugs and Medical devices (BfArM), following the “Guideline for Notification of a Compassionate Use Programme”, in accordance with the “Ordinance on Medicinal Products for Compassionate Use” (“Arzneimittel-Härtefall-Verordnung”, AMHV) in a study conducted at the Department of Internal Medicine 3 (Rheumatology and Immunology) of the Friedrich Alexander University Erlangen-Nurnberg, which consecutively enrolled five patients between 2021 and 2022. The rationale behind the use of this product without prior marketing authorisation was based on the features of the condition: rare and life-threatening, due to organ failures and lack of therapeutic options for achieving drug-free remission or even cure. Moreover, the disease tends to be refractory, with some patients not responding to the current state-of-the art therapies, and invalidating, as patients with SLE require lifelong treatment. B-cell depletion via anti-CD19 CAR-Ts has been thus considered a valid therapeutic strategy because it would have allowed the “reset” of the immune system, potentially resolving SLE. 

The study, whose interventions were reported to the relevant legal authorities (Paul Ehrlich Institute, PEI, Germany), demonstrated that MB-CART19.1 cell treatment did in fact led to depletion of B cells and disease remission, which notably did not require maintenance therapy afterwards.

On the other hand, it also showed that the CD19-targeted CAR T-cell approach may not be generalised to other autoimmune diseases: this approach requires diseases that not only are B cell-driven, but also develop on B-cell activation. Other autoimmune diseases exist that, despite resulting from B cells, are caused by long-lived plasma cells, which are usually CD19-negative [26], but successive iterations of CAR-engineered cells may bridge this gap in the long term. 

This could be the start of an important change in the treatment of this rare disease: the clinical effect of CAR-T-cell treatment is associated with resolution of the SLE patients’ autoimmunity, persisting even after about 100 days, when patients reconstituted their B cells. 

#### 2.1.4. CAR-T—Authorised Drug Combinations

The likeliest development in the near future regarding CAR-Ts clinical adoption and use is the combination of CAR-T cells with other drugs, already approved, mainly to enhance safety (Table 5). In the long term, combinations could be replaced by directly incorporating transgenes that allow CAR-T cells themselves to secrete drug-like moieties at their site of action. 

A major objective of the drug combination approach is to try to curb a crucial side effect of CAR-Ts, namely the Cytokine Release Syndrome (CRS).

From murine models replicating CRS, cytokines implicated in its development were identified, the main ones being GM-CSF, IL-1, IL-6.

Since the Granulocyte Monocyte Colony Stimulating Factor (GM-CSF) is a myeloid cytokine, it was hypothesised that CRS could be prevented from occurring [27] by reducing its concentration. Initially, the tests revolved around the combination of Lenzilumab, a GM-CSF-neutralising antibody, with anti-CD 19 CAR-Ts. This combination was compared to a baseline treated only with said CAR-Ts and it was observed that mice receiving the combination did not lose weight and had significant reduction of cytokines associated with development of CRS. The replication of these findings in humans was then attempted in a phase 1/2 clinical study (NCT04314843) of Lenzilumab use with Axicabtagene Ciloleucel, in patients with relapsed or refractory DLBCL. Six (6) patients across ten clinical centres have been successfully treated.

Recently, Yi and colleagues used GM-CSF knock-out CAR Ts at the clinical level [28], treating three (3) patients. Reportedly, none of these patients developed neurotoxicity, somewhat corroborating the previously reported findings. 

Interleukin-1 (IL-1) has an important role in severe systemic inflammation, and its association with CAR-T toxicities has been widely described. IL-1 as a target in CAR-T therapy was hit by administering Anakinra, an IL-1R antagonist [29] already authorised, in combination with the anti-CD19 CAR-T. Anakinra may be a useful adjunct to steroids and tocilizumab in the management of CRS and/or steroid-refractory ICANS resulting from CAR-T-cell therapies. However, prospective studies are needed to determine its efficacy in these settings.

Another therapeutic approach under investigation to control the CRS consists of the administration of a combination of CAR T cells with Dasatinib, at a critical time during the onset of CRS [30]. Dasatinib is already approved against Philadelphia chromosome-positive (Ph+) chronic myeloid leukaemia (CML), and in combination with CAR-T cells, it is expected to act by suppressing T-cell activation via inhibition of proximal T-cell receptor (TCR)-signalling kinases, such as Src, Fyn, and Lck [31]. This approach has already been documented in in vitro and mice models, and the resulting diminished reactivity of T cells had no bearing on their therapeutic efficacy. 

## 3. CAR-T Current Challenges

From clinical trials results on liquid or haematologic tumours, it is known that the majority of patients treated with CAR-Ts do have important responses, yet most of these are still not durable. 

This is not the case in solid tumours, where current CAR-Ts are not significantly effective, making it an area of unmet need despite being the focus of much of the current research in the field. 

CAR-T cells appear to be associated with significant toxicities of inflammatory nature, not only the cited Cytokine Release Syndrome (CRS), but also the Immune effector Cell-Associated Neurotoxicity Syndrome (ICANS), as well as cytopenias which then may lead to opportunistic infections. 

On the other hand, the development of allogeneic CAR-engineered cells, including non-T immune cells like Natural Killer (NK) and Macrophage (M), likely represents the largest gap in knowledge in the field today, which will require substantial investments to reach the clinic, although several reports of CAR-NK clinical trials are already available [5]. 

**Figure 3:**
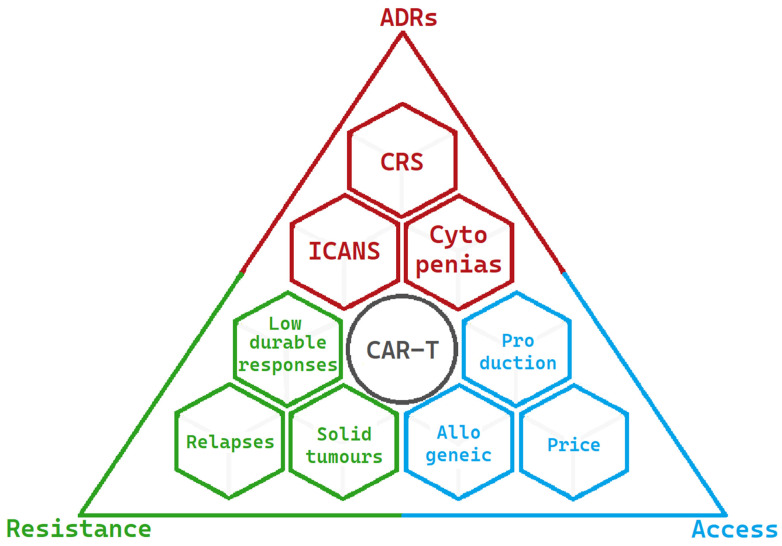
Limitations and challenges of CAR-Ts at present are summarised into three main areas. The most significant adverse drug reactions are in red, the factors resulting in resistance to CAR-T activity are in green, and the factors limiting access to CAR-T-cell therapy are in blue.

The present limitations of CAR-T-cell therapy are summarised in Figure 3:

### 3.1. CAR-T Target Choice Impacts Efficacy and Safety

Target choice can be considered a key element in the development of CARs: by choosing an ideal target, the safety of these products can be raised to high standards. The importance of a CAR’s target cannot be overstated, as it ties in directly to both efficacy and safety of these treatments.

Even if there is a vast and growing body of literature and expanding research, both preclinical and clinical, dealing with the discovery and testing of novel targets for CAR constructs [32,33], “target mining” or the search for more and better targets for a CAR’s application remains a significant gap in knowledge.

### 3.2. Adverse Drug Reactions

While some of the toxicities associated with CAR-T-cell therapy were anticipated (like the B-cell aplasia in anti-CD19 CARs due to its presence on healthy B cells), the two adverse events which have become distinctive of CAR-T-cell therapy were not anticipated from the early murine studies, i.e., CRS and ICANS [34]. 

#### 3.2.1. Cytokine Release Syndrome (CRS)

CRS usually develops 3–5 days after infusion, while ‘Immune effector-Cell Associated Neurotoxicity Syndrome’ (ICANS) starts 5–7 days after infusion and is likely connected to CRS development. 

CRS is caused by extremely high levels of cytokines due to a strong immune response. Typical CRS symptoms range from sustained fevers, hypotension, and in general, the need for airway protection, low blood pressure, high fevers, and circulatory failure, which might require the patient’s admission to the Intensive Care Unit (ICU) and the use of ventilators.

This implies that a small clinical centre, not equipped with advanced facilities, will not be authorised for the use of CAR-Ts, thus contributing to a reduced access to this therapeutic opportunity. 

Recent studies in cytokines involvement in CAR-T therapy’s CRS show that many cytokines derive from the CAR-T treatment. This occurs as engineered cells proliferate in vivo when activated by coming in contact with tumour cells, and produce dramatic increase in cytokines. Moreover, some key cytokines mediating CRS, like IL-6, are produced by Tumour-Associated Macrophages (TAMs), myeloid-type cells found in the patient’s tumour [35,36]. 

A direct proportionality between the tumour burden, the scale of T-cell activation and action, and the severity of the syndrome has been observed. As a result, the most fragile segment of patients, those with more severe disease conditions, tend to suffer the direst CRSs.

Despite incremental improvement in the ability to recognise Cytokine Release Syndrome and the early treatment with monoclonal antibodies that might induce IL-6 blockade (i.e., Tocilizumab), deaths linked to this side effect continue to be observed. Thus, a deeper understanding of CRS, its causes and mechanism of actions, is needed to control, treat, or reduce occurrence in future CAR-T medicinal products.

#### 3.2.2. CAR-T-Mediated Neurotoxicity (ICANS)

ICANS appears to be due to the expression of CD19 on some neurons and blood–brain barrier lining. ICANS main symptoms are confusion, aphasia, seizures, and encephalopathy.

Even if, according to MRI observations during neurotoxicity, these symptoms tend to be transient, ICANS-induced encephalopathy can be deadly if associated with brain edema, and high-grade ICANS can result in the destruction of the blood–brain barrier (BBB) [37], with potential infiltration of T cells, B cells, and myeloid cells into the Cerebral Spinal Fluid (CSF) and the Central Nervous System (CNS). 

GM-CSF seems to play a central role in CAR-T-mediated neurotoxicity, as demonstrated by the analysis of some of the 2017 pivotal clinical trials [38]. 

In addition, the infiltration into the CSF of CD14-positive cells and monocytes, like Tumour-Associated Macrophages (TAMs), has been associated with Grade ≥ 3 neurotoxicity, thus suggesting a role for these cells as a target for preventing ICANS. Currently, the treatment of ICANS includes solely the administration of corticosteroids and supportive care. In addition, since ICANS often occurs with CRS, the resolution of the former is needed for a proper CRS treatment; however, the spontaneous resolution can only happen if ICANS is low-grade, while severe ICANS (grade ≥ 3) remains a critical condition, thus highlighting a gap in knowledge and the need of finding new and resolutive treatments. ICANS and neurotoxicity are obviously crucial concerns to the application of CAR-Ts in the neurological field. Through the observation of haematologic patients with known CNS involvement, presence of MRI changes or baseline neurological disorders have been linked to increased risk of ICANS [39]. In recent years, several antigens were identified as targets for CAR-T-cell therapy against primary brain tumours (e.g., GBM) which are currently in early clinical development in the US and China [40,41]. 

### 3.3. Resistance to CAR-T Activity

Resistance to CAR-T activity is a multifactorial outcome due to different aspects, mainly related to patients’ cells used to generate the CAR-Ts, T-cell exhaustion and tumour microenvironment (TME).

Regarding the influence of starting materials in autologous treatments, patients T cells’ functioning could be defective even before the transduction of T cells with CARs. This seems to be the case in heavily pre-treated patients, who approach CAR-Ts as a third line of treatment (e.g., patients who could benefit from CAR-T treatment may not be eligible because of low WBC counts and/or activity levels), and it is one of the reasons for the interest in allogeneic CAR-Ts. For similar reasons, these therapies have been moved to earlier lines of treatment, like in the case of the Zuma-7 trial (NCT03391466), a comparison trial which allowed Yescarta© to be the first CAR-T to ever be available as second-line treatment, for HGBL and DLBCL.

Defective T-cell function may also appear as a result of genetic modification. This specific form of decline in functions is known as ‘T-cell exhaustion’, a poorly understood continuous differentiation process, in which T cells are transformed from precursors to terminally differentiated T cells, thus losing cellular functionality.

The tumour microenvironment (TME) plays a critical role in the response to tumour treatments. Suppression exerted by the tumour’s TME can down-regulate activity of both CAR-Ts and physiological T cells. CAR-T inhibition mediated by the TME in solid tumours is a major area of study [42]. Currently, remissions in solid tumour indications after CAR-T infusion remain few and short-lived. This may be due to the difficult trafficking to the tumour. In several studies, CAR-Ts were able to get inside the tumour mass, although they were reported to be inhibited at the site. This inhibition is mediated by the immunosuppressive TME, and solving such inhibition could increase the number of possible new medicinal products for new and critical indications in the solid tumour space.

Among the approaches to TME-mediated resistance, dual-targeting CAR-Ts may be used to stop Cancer-Associated Fibroblasts (CAFs) from inhibiting the CAR-Ts. CAFs are a cellular component of the TME, well documented, for example, in multiple myeloma, which can inhibit BCMA CAR T cells [43] such as Abecma (Ide-Cel). A dual CAR targeting BCMA as well as specific CAF antigens such as FAB may allow CAR-Ts to deplete this source of inhibition while carrying out their anti-tumour activity.

Another approach to overcome TME lies in the CAR design called TRUCK CAR Ts, used against inhibitory extracellular vesicles. Such vesicles are part of the milieu of the TME and have been documented [44] expressing PD-1L, thus blocking CAR-Ts immune checkpoints, or even containing inhibitory-micro-RNAs (miRNAs), which can target T-cell pathways resulting in T-cell exhaustion. A CAR able to allow its T cell to secrete FAS-L2 to target such miRNAs, or even PD-1L inhibitor-like moieties may be able to overcome inhibition from the TME’s extra-cellular vesicles.

Lastly, another component of the TME for which potential solutions have been investigated is the cytokine Transforming Growth Factor-β (TGF-β), which has a prominent immunosuppressive role in the TME [45]. An approach that has already been clinically investigated is the development of a CAR armoured with a TGF-β Receptor Dominant-Negative (RDN) [46] that would keep expanding despite TGF-β’s immunosuppressive signal. 

Novel designs, such as peptide-centric (PC) CAR-Ts [47], are possible solutions to this type of roadblock.

### 3.4. Access to CAR-T-cell therapy

The number of patients having access to CAR-T-cell treatment is currently quite limited, despite the high and increasing level of interest, investments, and research in the field. According to the EBMT registry [48], 2500 patients were infused with a CAR-T medicinal product in 2021. When comparing this figure to the total number of patients for diseases where at least one CAR-T product has that indication [49], they only represent about 1% of the total number of cases. Crucial roadblocks to the increase of CAR-T-cell therapy access to patients are the long and complex manufacturing processes and high costs.

Although the actual CAR-T-based treatment price can vary significantly among EU member states, it may indicatively be considered at around EUR 350.000, with possible variations due to different agreements stipulated between the Marketing Authorization Holders (MAHs) and member states’ NHSs. 

Despite this very high price tag, in principle making CAR-Ts profitable products, it crushes their ability to fully become part of the mainstream cancer treatment options. 

Two potential solutions have been discussed in the available literature, consisting of (i) increasing production outputs to lower costs (e.g., increased levels of automation and miniaturisation of GMP-grade production facilities), and (ii) manufacturing of allogeneic products to lower costs (e.g., development of non-T CAR-engineered cells, like CAR NK and CAR M and/or the use of novel sources of biologic materials, ranging from healthy donors to iPSCs, generating so-called iCAR-Ts). The first is reasonably the near-term solution that can be expected in the coming years, while the second can constitute instead a long-term objective. Allogeneic products will allow a virtually unlimited quantity of medicinal products, with lower costs and the possibility to increase the complexity, and thus hopefully the effectiveness of CARs, by introducing multiple transgenes and/or mutations to the engineered cell. 

#### 3.4.1. GMP Cell Manufacturing: Cost and Complexity of Production

**Figure 4:**
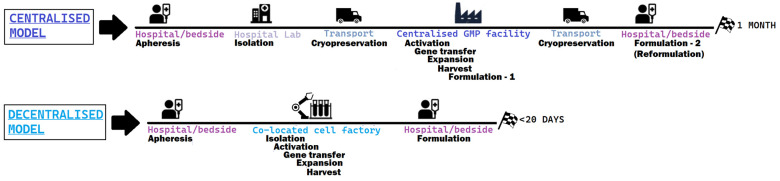
Steps of the prototypical centralised model, the one adopted by authorised CAR-T products versus a decentralised model, much more compact and less variable.

When analysing and comparing the efforts to address the cost and complexity of CAR-T-cell therapy, a recurrent theme across both literature and scientific institutions developing this technology is the need to pass from a centralised process to a decentralised production process [50,51], both shown in Figure 4:

In a centralised model, the key to production is the company and its GMP facilities. Current challenges in access to therapy partly stem from the complex nature of the process, that can take between 3–4 to 6–8 weeks from the time that leukapheresis is performed and patient’s cells are shipped to the company, and the product is back to the hospital for being reinfused. The extremely expensive nature of this kind of medicinal product, due to this complex production process as well as the low scale of production, translates to the fact that only major academic centres can afford this therapeutic option, further decreasing access, similarly to the established third-line treatment alternative to CAR-Ts (bone marrow transplantation), which is not available in small clinical centres. In addition, by looking at the clinical trial results with the centralised model, it was observed that some patients were invariably lost because the time to acquire the medicinal product was too long for the patient to wait, and the bridging therapy was not sufficient.

On the other side, the decentralised production model aims at significantly reducing the overall time to reach the patient, by cutting storage and transportation times through the use of mobile cell factories. The possibility of doing CAR-T infusions as an outpatient therapy, an increase in the number of centres that can adopt CAR-T-cell therapies, shortening the time for potentially life-saving innovations to reach the clinical setting, are some of the potential advantages of this approach. A nice example of a decentralised and flexible approach was tested in St. James’s Hospital and the Trinity College Dublin Clinical Research Facility in Dublin (July 2022), moving a cell factory closer to patients using a mobile GMP facility for CAR-T [52].

Further investigations into the use of more automated manufacturing platforms and co-located GMP-compliant facilities are needed. Examples of currently ongoing innovative developments in the field are carried out by several players, such as Lonza pharmaceuticals^®^ [53], Mylteni therapeutics^®^ [54], and aCGT Vector [55]. 

The establishment of GMP manufacturing quality standards is considered an important challenge with regulatory implications. Specific guidelines do not exist yet, and coordination between regulators and manufacturers is needed to bridge this gap in the near future.

#### 3.4.2. Allogeneic CAR-T

Another sprawling field of interest which addresses the limitations of CAR-T-cell therapy are “Off-the-shelf” CAR-Ts, obtained from healthy donors (allogeneic T cells), which can provide high amounts of fully functional cells and allow multiple CAR-T-cell products to generate. Such an approach may increase patients’ access to therapy, also reducing the delivery time of products that can be stored like conventional biologics.

Their major limitation is the need to undergo an additional editing step to prevent Graft-versus-Host (GvHD) disease-type rejection [56]. Though results are inferior to the autologous CAR-Ts at the moment, the presence of therapeutic action proves the potential of this platform. Along with T cells engineered to be allogeneic CAR-Ts, Natural Killer (NK) cells have been successfully used as a separate cell type to produce allogeneic CAR-engineered medicinal products, with no relevant toxic effect [57].

Another promising approach for the generation of allogeneic CAR-T products foresees the use of in vitro-induced pluripotent stem cells (iPSCs) [58], instead of patients’ blood cells from healthy donors. 

The promise of these engineered stem cells is fascinating: with a potentially perpetual supply of virtually unlimitedly editable cells, the versatility of iPSC-derived CAR-T cells (iCAR-Ts) has garnered increasing investments and interest. iPSC technology is currently in its “first wave” and suffers from several limitations, like the absence of a clear regulation for the product’s quality assessment and release for clinical use, the lacking acceptance of key stakeholders towards iPSC technology, especially patients, and the non-availability of robust and scalable manufacturing protocols for clinical-grade iCAR-T cells.

Other gaps in knowledge that are likely to impact in the long term the access to iCAR-T therapies are the suboptimal function and developmental maturity of iPSC-derived CAR-Ts in comparison to ‘primary’, autologous CAR-Ts. So far, studies conducted on T cells derived from iPSCs (TiPSCs) only managed to produce CD8αα CAR-T cells with a low activity profile, similar to innate T cells [59]. This phenomenon is most probably attributable to the β-selection stage of T-cell development, which seemed to be skipped. This can deprive CD8+ cells of important moieties, such as CD28 and CCR7, respectively, a co-stimulatory receptor which allows significant activation and survival of T cells, and a memory marker which characterises the central memory phenotype, hence leading to significant persistence in vivo [60].

Currently, to use TiPSCs as raw material to engineer CAR-Ts, a master iPSC cell bank must be created via gene editing and subcloning, specifically dedicated to the disease in study. This is a long and technically challenging, substantially expensive process, which can generate genotoxic products, and it prevents the introduction of more complex CAR designs (armoured or dual CARs). Surpassing this technological challenge appears to be crucial to the ability to scale up quantities of CAR-T produced starting from iPSCs, and the potential of iPSCs to compete with cells from healthy donors. 

## 4. Conclusions

Gene therapies hold a big promise for oncologic patients as well as rare disease patients. The analysed areas for improvement preventing a faster uptake and consolidation of the use of such therapies, and that would require an update in the regulatory approaches, deal with three main aspects: (i) the regulatory approval landscape, which may be overcome by adopting regulatory assessment based on real-world data (RWD) to accelerate approvals; (ii) the manufacture and release model, which could be improved by introducing decentralised (bedside) manufacture to reduce production time and eliminate human variability in case of automated equipment; and (iii) the reimbursement model, which can be approached using the Quality Adjusted Life Years (QALY) shaped for CAR’s Patients [61,62].

These fields (approval, manufacture, and reimbursement, respectively) operate based on conventional pharmaceutical models, which slow down access to ATMPs.

In the long term, roadblocks that need to be overcome to achieve widespread cell therapy adoption should primarily include standardising cell therapy production and removing transportation hurdles, like the need for default freezing/thawing and transportation of human cells: this introduces risks to cell viability and potency as well as delayed treatment. Working directly with innovators is needed to develop next-generation cell therapies in-hospital, for example, the so-called “GMP-in-a-POD” platforms [55], and lastly, to provide access to multiple cell engineering technologies appropriate to the cell modality. 

Innovations addressing the high cost and the long manufacturing duration of CAR-T-cell therapy will have a tremendous societal and economic impact, and developing the procedures and tools to efficiently produce off-the-shelf, TiSPC-derived CAR-Ts is key to both endeavours. 

All approved CAR-T-cell products are orphan medicines, authorised for rare diseases. They present several recurrent challenges regarding efficacy, safety, and access that have been only partially solved. Among those challenges identified by this review, the low efficacy in solid tumour, runaway inflammatory side effects, and difficult and expensive manufacturing are considered priorities in the field and must be addressed by regulators and scientists.

From the regulatory point of view, this review proposes that the EU regulatory framework is going to require updates to properly oversee such innovative therapies and the challenges they pose. However, the analysis of the framework as is already shows several possible routes to allow the access to patients suffering from rare diseases.

From the scientific point of view, CAR technology holds a great promise. This is attested by recent indications’ expansions for the approved CAR-T products, by the encouraging results in the clinical setting, and finally, by a technological basis that is growing in the direction of increasing production while lowering cost, which constitutes a critical need. 

The ongoing research in the field of CAR-T-cell therapy, such as developments in the sustainable and safe use of advancements in gene editing techniques to achieve multiplex editing (introducing multiple mutations on the engineered cells), shows potential scenarios for future breakthroughs in their clinical applications, and in creating novel sources of starting cellular materials. At the same time, even if gene editing of CAR T cells is limited to somatic cells, ethical aspects regarding modification of primary lineages and off-target editing will need to be addressed from a scientific and regulatory point of view before the full application of this technique can be transferred to patients. The resulting production of more innovative as well as allogeneic products able to avoid graft-Vs-host disease could reshape treatment of several oncologic indications, and even give hope to develop treatments for many diseases still incurable today.

## Figures and Tables

**Table 1:** Authorised CAR-T-cell therapies within the European Union and their features.

Commercial Name Active Principles Company	Product Features: Generation Origin Target	Initial Indications (EU)	Indications Today (05/2023) (EU) (US) *	Approval Date	Orphan Designation
Kymriah™ Tisagenlecleucel **Novartis**	2nd Gen Autologous CD19	B-ALL, DLBCL	B-ALL, DLBCL, FL, HGBL *	US: 30 August 2017 EU: 23 August 2018	Yes
Yescarta™ Axicabtagene ciloleucel **Kite—a Gilead company**	2nd Gen Autologous CD19	PMBCL, DLBCL	PMBCL, DLBCL, HGBL, FL	US: 18 October 2017 EU: 23 August 2018	Yes
Tecartus™ Brexucabtagene autoleucel **Kite—a Gilead company**	2nd Gen Autologous CD19	Mantle Cell Lymphoma	Mantle Cell Lymphoma, B-ALL	US: 24 July 2020 EU: 14 December 2020	Yes
Abecma™ Idecabtagene vicleucel **Bristol-Meyers Squibb**	2nd Gen Autologous BCMA	Multiple Myeloma	Multiple Myeloma	US: 26 March 2021 EU: 18 August 2021	Yes
Breyanzi™ Lisocabtagene maraleucel **Bristol-Meyers Squibb**	2nd Gen Autologous CD19	FL3B, PMBCL, DLBCL	FL3B, PMBCL, DLBCL, HGBL *	US: 05 February 2021 EU: 4 April 2022	Yes
Carvykti™ Ciltacabtagene autoleucel **Janssen-Cilag international**	2nd Gen Autologous BCMA	Multiple Myeloma	Multiple Myeloma	US: 28 February 2022 EU: 25 May 2022	Yes

**Table 2:** All the CAR-T medicinal products (8) that have benefitted from the PRIME scheme in the MA evaluation and have been authorised or withdrawn (as of 3 May 2023).

Name	Therapeutic Indication	Date of Granting PRIME Eligibility	Status
Axicabtagene ciloleucel (Yescarta)	Treatment of adult patients with diffuse large B-cell lymphoma (DLBCL) who have not responded to their prior therapy, or have had disease progression after autologous stem cell transplant (ASCT)	26 May 2016	Authorised, see EPAR
Tisagenlecleucel (Kymriah)	Treatment of paediatric patients with relapsed or refractory B-cell acute lymphoblastic leukaemia	23 June 2016	Authorised, see EPAR
Autologous CD3+ T Cells Expressing CD19 Chimeric Antigen Receptor (JCAR015)	Treatment of relapsed/refractory adult B-cell Acute Lymphoblastic Leukaemia (ALL)	15 September 2016	PRIME eligibility withdrawn at the request of the applicant (development discontinued)
Lisocabtagene maraleucel (JCAR017) (Breyanzi)	Treatment of relapsed/refractory diffuse large B-cell lymphoma (DLBCL)	15 December 2016	Authorised, see EPAR
Idecabtagene vicleucel (bb2121) (Abecma).	Treatment of relapsed and refractory multiple myeloma patients whose prior therapy included a proteasome inhibitor, an immunomodulatory agent, and an anti-CD38 antibody	9 November 2017	Authorised, see EPAR
Autologous peripheral blood T cells CD4 and CD8 selected, and CD3 and CD28 activated transduced with retroviral vector encoding an anti-CD19 CD28/CD3-zeta chimeric antigen receptor and cultured (KTE-X19) (Tecartus)	Treatment of adult patients with relapsed or refractory mantle cell lymphoma	31 May 2018	Authorised, see EPAR
Autologous human T cells genetically modified ex-vivo with a lentiviral vector encoding a chimeric antigen receptor (CAR) for B-cell maturation antigen (BCMA) (JNJ-68284528) (Carvykti)	Treatment of adult patients with relapsed or refractory multiple myeloma, whose prior regimens included a proteasome inhibitor, an immunomodulatory agent, and an anti-CD38 antibody and who had disease progression on the last regimen	28 March 2019	Authorised, see EPAR
Autologous CD4+ and CD8+ T-cell populations transduced with a genetically engineered replication-incompetent, self-inactivating lentiviral vector to express a BCMA-specific CAR (JCAR125)	Treatment of relapsed/refractory multiple myeloma whose prior therapies included autologous stem cell transplant if they were eligible, a proteasome inhibitor, an immunomodulatory agent, and an anti-CD38 antibody	14 November 2019	PRIME eligibility withdrawn at the request of the applicant (development discontinued)

**Table 3:** List of CAR-T medicinal products currently in the PRIME scheme.

Name	Target Antigen	Origin	Therapeutic Indication	Date of Granting PRIME Eligibility
ARI-0001 (CART19-BE-01)	CD19	Autologous	Treatment of patients older than 25 years with relapsed/refractory acute lymphoblastic leukaemia	21 July 2016
MB-CART2019.1	•CD19 •CD20	Autologous	Treatment of patients with relapsed and refractory diffuse large B-cell lymphoma (DLBCL) after frontline therapy and who are ineligible for autologous stem cell transplantation	19 September 2019
letetresgene autoleucel (Lete-Cel) (GSK3377794)	NY-ESO-1	Autologous	Treatment of HLA-A*0201, HLA-A*0205, or HLA-A*0206 allele-positive patients with inoperable or metastatic synovial sarcoma who have received prior chemotherapy and whose tumour expresses the NY-ESO-1 tumour antigen	17 October 2019
ADP-A2M4	MAGE-A4	Autologous	Treatment of HLA-A*02-positive patients with inoperable or metastatic synovial sarcoma who have received prior chemotherapy and whose tumour expresses the MAGE-A4 tumour antigen	23 July 2020
BNT211	Claudin6	Autologous	Treatment of testicular germ cell tumours	17 September 2020
CD30.CAR-T	CD30	Autologous	Treatment of classical Hodgkin lymphoma	25 March 2021
CT041 (CAR-CLDN18.2)	Claudin18.2	Autologous	Treatment of patients with advanced gastric cancer who have failed at least two prior lines of systemic therapy	11 November 2021
CT053	BCMA	Autologous	Treatment of patients with relapsed and/or refractory multiple myeloma (MM) whose prior regimens included a proteasome inhibitor, an immunomodulatory agent, and an anti-CD38 monoclonal antibody	16 December 2021
Obecabtagene autoleucel (Obe-Cel) (AUTO1)	CD19	Autologous	Treatment of relapsed or refractory B-cell acute lymphoblastic leukaemia	23 June 2022

**Table 4:** CAR-T cell-based treatments authorised in Italy under HE (Source Database AIFA-Istituto Superiore di Sanità, Italy).

			**2021**			
**Age**	**Target**	**Derived From**	**Medicinal Product**	**Treatment Outcome**	**Degree of Disease**	**Donor**
18	ALL-B	HSCT	Allogeneic CD19 CAR-T cells	Unknown	Risk of recurrence	HLA identical
2	ALL-B	HSCT	Allogeneic CD19 CAR-T cells	Unknown	Resistant to chemotherapy	HLA identical
11	ALL-B	HSCT	Allogeneic CD19 CAR-T cells	Unknown	Multiple relapses and resistant	HLA identical
20	ALL-B	HSCT	Allogeneic CD19 CAR-T cells	Unknown	Refractory to multiple treatments	HLA identical
6	ALL-B	HSCT	Allogeneic CD19 CAR-T cells	Positive	Relapse	HLA identical familial
29	ALL-B	HSCT	Allogeneic CD19 CAR-T cells	Unknown	Fifth time reoffend	HLA identical
			**2022**			
**Age**	**Target**	**Derived from**	**Medicinal Product**	**Treatment Outcome**	**Degree of Disease**	**Donor**
11	Neuroblastoma	HSCT	Allogeneic GD2 CAR-T cells	Unknown	Multi-treated and relapsed	HLA identical
12	ALL-B	HSCT	Allogeneic CD19 CAR-T cells	Unknown	Relapsed for the third time	HLA identical
8	ALL-B	1°maintenance therapy	Allogeneic CD19 CAR-T cells	Unknown	Relapsed for the first time	HLA identical
17	ALL-B	HSCT	Allogeneic CD19 CAR-T cells	Positive	Relapse	HLA identical
4	ALL-B	1°maintenance therapy	Allogeneic CD19 CAR-T cells	Positive	Relapsed and associated with Down syndrome	HLA identical
26	ALL-B	HSCT and autologous CAR-T therapy	Allogeneic CD19 CAR-T cells	Positive	Relapse	HLA identical
8	Neuroblastoma	HSCT	Allogeneic GD2 CAR-T cells	Positive	Recurrent, metastatic, and refractory	HLA identical
9	ALL-B	HSCT	Allogeneic CD19 CAR-T cells	Positive	Relapsed	HLA identical intrafamilial
33	ALL-B	HSCT	Allogeneic CD19 CAR-T cells	Positive	Relapsed	HLA identical
6	Neuroblastoma	HSCT	Allogeneic GD2 CAR-T cells	Unknown	Metastatic	HLA identical familial
34	ALL-B	HSCT	Allogeneic CD19 CAR-T cells	Unknown	Relapse	HLA identical familial
			**2023**			
**Age**	**Target**	**Derived from**	**Medicinal Product**	**Treatment Outcome**	**Degree of Disease**	**Donor**
27	ALL-B	HSCT from MUD	Allogeneic CD19 CAR-T cells	Positive	Relapse	HLA compatible (MUD)
5	Neuroblastoma	Chemotherapy and autologus CS reinfusion	Allogeneic GD2 CAR-T cells	Unknown	Metastatic and refractory	
8	ALL-B	HSCT	Autologous CD19 CAR-T cells	Positive	Relapse	HLA identical familial
4	Neuroblastoma	Second-line chemotherapy	Autologous GD2 CAR-T cells	To be defined	Recurrent, metastatic, and refractory	
8	Lupus Erythematosus	Worsening of the clinical condition	Autologous CD19 CAR-T cells	To be defined	Systematic and resistant	
9	Neuroblastoma		Autologous GD2 CAR-T cells	To be defined	Recurrent, metastatic, and refractory	
10	Neuroblastoma		Autologous GD2 CAR-T cells	To be defined	Recurrent, metastatic, and refractory	
7	Neuroblastoma		Autologous GD2 CAR-T cells	To be defined	Recurrent, metastatic, and refractory	
6	ALL-B	HSCT	Allogeneic CD19 CAR-T cells	To be defined	Relapse	HLA identical

**Table 5:** Among solutions to CAR-T limitations which are likely to get to the clinic in the near future, combinations of authorised drugs with CAR-Ts are an important development to curb toxicities.

Cytokine Causing CRS	Product Used in Combination
Granulocyte Monocyte Colony Stimulating Factor (GM-CSF)	Lenzilumab
Interleukin 1 (IL-1)	Anakinra
Proximal TCR Signalling Kinases, (Lck, etc.)	Dasatinib

## Data Availability

Data are publicly available.
